# Preventive Treatment of Urinary Calculi

**Published:** 1889-11

**Authors:** B. F. Church

**Affiliations:** Austin, Texas


For Daniel's Texas Medical Journal.

### ON THE PREVENTIVE TREATMENT OF URINARY CALCULI.

BY B. F. CHURCH, M. D., AUSTIN, TEXAS.

Read before the Austin District Medical Society, June 20, 1889.

WHILE the origin of renal calculi is not as yet perfectly understood, the study of the subject by eminent observers has by no means been neglected. Facts deducted from these investigations are constantly broadening the avenue to rational preventive treatment. We are not in a position, though, to foretell a stone formation, for all of the conditions may seem to be present, and none occur; on the other hand, a stone is sometimes found when least expected.

Preventive treatment is generally first thought of after a patient has passed a renal concretion. There is doubtless a better course to pursue, than subject them to constitutional treatment, unless it is otherwise indicated, for the various so-called diathesis in anticipation of the disease, it being very important that we differentially diagnose a conglomeration of crystals, single crystals—properly termed gravel, within itself harmless,—and a *true concretion*. The latter may be smaller than the crystals, or a cluster of crystals, but under the microscope show a laminated structure, which is one of its characteristics. If the careful and scientific researches of Rainey, Ord, and Carter are properly regarded, this distinction has a very important bearing upon the prognosis and treatment of culculus disease.

Crystals, under ordinary condition in the urine, do not unite to form a stone; something else is necessary and that entity is a colloid, which Rainey and Ord have demonstrated acts as a cement which binds the mass together. Ord says, "To make calculi of uric acid without colloids would be as hopeless a task as making ropes of sea sand." Thompson has called special attention to the long known fact that in a case of so-called phosphatic diathetic, an individual's urine may be white with precipitated phosphates, and a stone will not form as long as the man's membrane of the urinary tract is free from inflammation; the albuminoid or colloid to act as the cement, is lacking, which the mucopus—a result of an inflammation—would furnish. On the other hand, there may not be an abnormal quantity of crystals or amorphous phosphates present, when, persumably some solid body, as a coagula of blood, shreds of mucus, epithelium, or perhaps bacteria,—with the proper co-operation of the colloids normally present—becomes the neucleus, and a primary (acid) stone starts into existence, or the presence of a foreign body or other agent capable of exciting an inflammation of the mucous membrane of any part of the urinary passage—with an alkaline condition of the urine—may speedily give rise to a secondary (alkaline phosphatic) stone.

Ziemsen, in general terms, denies the causative influences of certain diatheses; at the same time does not seem to credit the *local condition* with the important role it deserves. He gives as causes for calculi: increased acidity of the urine, a diminished temperature, the presence of solid bodies, etc.

He says: "It would be quite incorrect to assert that uric acid concretions are always developed from constitutional causes."

It is a singular fact to note that all of the popular remedies used from time immemorial against urinary lithiasis have been some of the various forms of alkalies, whether they were obtained from calcined snail shells, as recommended by Pliny, the ashes of egg shells, various plants, insects, and reptiles of later times, or many of the Spas and patent nostrums so popular to this day. Believing that this line of treatment, first used empirically, like many of the old traditions, has a value, it is nevertheless true too much reliance is often placed on this class of remedies for the treatment of the disease in question. A plea shall also be entered against the use of stimulating diuretics.

Calculi are usually made up of several ingredients, one of which is always animal matter, a sort of stroma, that pervades the mass in which the other materials, crystalline and amorphons, are deposited; it is probably always of a uniform character, and contains mucus, fibrin, and albumin; but its exact composition is not known.

The other ingredients or ground work of a stone, as a rule, is composed of several materials, urates, uric acid, and oxalates occurring together in the same stone, and sometimes phosphates are added to the outside. It is evident that a calculus owes its existence to its nucleus. and for our purpose—that of preventive treatment—the name of a stone should be according to the composition of its nucleus, and only by determining its character can the special tendency of the patient toward calculus be decided, and possibly a more intelligent preventive treatment be instituted.

The classification of stone into two great classes, as recommended by Keys,* is probably the best:

"I. *Primary stone*,--the mucous membrane of the urinary tract being sound when the stone forms,—uric acid (urate of sodium patassium and lime,) oxalate of lime, crystalline phosphate of lime and indigo."

"II. *Secondary or symptomatic stone*,—the mucous membrane of the urinary passage being in a catarrhal state at the point where the stone forms,—urate of ammonium, amorphons phosphate of lime, mixed phosphate, or fusible calculus, and urastiath."

Different authorities estimate that from 80 to 90 per cent. of urinary calculi are of uric acid or urates. The next in frequency

---

*International Encyclopædia of Surgery.

is oxalate of lime. Both are closely allied chemically, products of acid urine, and usually found in the kidneys. Practically there is no distiction between the uric acid and oxalic acid diathesis, for chemists of reputation now agree that oxalic acid is one of the products of the oxidation of uric acid; therefore, oxalate of lime in large quantities in the urine signifies nothing else than the uric acid diathesis—or a state of the organism which Dr. Murchison describes, and properly designates lithæmia.

The other forms of primary stone, cystine, zanthine, etc., are so rare as to almost be looked upon as curiosities; practically then, the problem before us is: How may we best prevent the formation of uric acid calculi?

The identity of gout and uric acid gravel is unquestionable. Both are constitutional diseases and spring from the same root, though they constitute two different series of phenomena. It will be impossible to give a satisfactory explanation of lithæmia until we learn more cf the origin of uric acid in the body and of its metamorphosis. Many of the theories advanced seem very plausible, but from the lack of demonstration, none can be unquilifiedly accepted. Heredity seems to be the most important foctor in the etiology of the gouty diathesis; next comes the use of wines and malt liquors and a diet rich in the hydro-carbonaceous substances.

Garrod believes that the uric acid excreting function of the kidneys may be suspended, or completely lost, without the loss of power to eliminate other waste products. Heidenhein and his pupils have shown that the kidney is not a mere mechanical filter, but an organ endowed in its different parts with various selective actions, thus: while the epithelium which lines one part of the uriniferous tubules are separating one waste product from the blood, that of another part eliminates some other. This epithelium, as a whole or in part, has times of inactivity and excitation, and as its period of activity is increased or diminished, we find more or less marked disturbances supervening in the economy. This disturbance of the excreting functions of the kidneys is well marked in gout, and the excess of uric acid remaining in the blood is vicariously deposited in the tissues.

Not raising the question whether there is an abnormal quantity of uric acid produced in the system during an attack of lithiasis, the fact remains that there is an excess in the urine, showing that the kidneys are performing their functions, in this direction at least, and do not need stimulating by diuretics.

In suitable cases, the action of alkalies may be described as reducing the irritability of the mucous membrane of the urinary tract by their power to correct the acidity of the urine, thereby preventing a catarrh, with its concomitant muco-pus formation, which may furnish the colloid for a primary stone, or the ammoniacal changes essential for phosphatic accretions; and, if carried further, until the urine is decidedly alkaline, would dissolve uric gravel, and possibly small concretions, (if they were not oxalic acid or the oxalates,) clearing up the patient's urine, and giving immediate relief. The question may be asked: What more can be desired? A great deal; the source of the evil has not been reached. The enemy has only been made to disappear from view, but is not driven from the field, and is sure to appear again when the treatment is discontinued.

Dujardin Beaumetz, and other authorities, claim that the alkalies do not act altogether by the power of neutralizing the uric acid in the body, but they "Energize the phenomena of oxidation of the economy, and thereby aid the transformation of uric acid into urea." Granting they do augment oxidation, it does not prove they aid the transformation of uric acid into urea. The theory which has long been held—and supported on physiological grounds—that the proteids of the body were first oxidized to uric acid and subsequently to urea, and that the former was but imperfectly oxidized urea, is not tenable. Birds have a higher temperature than mammals, and their blood is highly oxidized, yet they excrete little or no urea, but uric acid instead. It should be remembered that the long continued use of alkalies will cause anæmia, and that the "alkaline cachexia" described by Trousseau—which he believed to be the result of the deglobulizing action of alkalies—is not altogether a myth. If soda should be the one used, and from the fact that its salts abound in the blood serum, and the salts of potash predominate in the corpuscles, Gulber shows that to greatly augment the quantity of soda in the serum, the blood globules lose their potassa, and in consequence their hæmatic properties.

In the treatment of the uric acid diathesis, two distinct problems are to be considered: first, will a temporary removal of the symptoms suffice—bridging over a time of danger? second, has the patient strong hereditary influences, passed a renal concretion, or does his environments constantly act as a predisposing cause?

The alkaline treatment is obviously indicated for the first condition, and should be exhibited during the third hour after eating, at the time when the acid in the chyme is neutralized, or has been absorbed. Benzoic acid may be given best in the form of benzoate of soda, as a part of this substance is transformed—if the liver is not seriously affected—into hippuric acid, and eliminated by the kidneys, which destroys the uric acid present in the urine, and converts the insoluble urates into soluble hippurates.

If the second, all means should be employed to encourage the physiological action of those organs that minister to perfect assimulation. A superabundance of uric acid in the body is doubtless due to some defective action of the liver, aided by the skin and other emunctories, except the kidneys, inefficiently performing their functions. Remedies should be directed to the primæ viæ. For their stimulation by medicinal agents, in plethoric patients, the judicious use of mercury with the natural mineral waters; those which contain principally sulphate of soda and magnesia, as Friedrichshall, Hunyandi, Janos, etc., deserve the first rank. These saline aperients, among other beneficial effects, promote the removal of mucus from the alimentary canal, and thus check the development of lactic and butyric acids.

The value of sulphate of soda requires special mention. It is not only a good cholagogue, but a part of its sulphuric acid is eliminated by the urine in a free state, and may combine with some of the bases there and form a sulphate which is soluble. Theoretically, sulphur, sulphates, or sulphurettes, are indicated whin the urine is ammoniacal. Glauber's salt, with a slight addition of sulphate of magnesia, is a good substitute for the more expensive mineral waters.

Sir Henry Thompson claims that an undue uric acid deposit can be made to disappear in nineteen cases out of twenty by a proper dietetic regulation.

Since uric acid, like urea, contains a large proportion of nitrogen, and "is found within the body by the metamorphosis of nitrogenous organic substances," (Halton,) it was the popular belief that this element (nitrogen) should be eliminated as far as possible from the dietry. As already mentioned, this view of the matter will not furnish the secret of successful treatment. It is the fats, sugars and starches that should be proscribed. Probably no harm would come from a moderate use of the recently discovered product, saccharin, for sweetening purposes, if the loss of sugar is greatly felt. Fresh green vegetables are especially to be recommended. By their free use the urine can be made to resemble that of the herbivora in that it contains a large proportion of hippuric acid in place of uric. Of the fruits, apples are perhaps the most wholesome; in fact, it is claimed that cider is a good remedy for stone. The sweet fruits, and nearly all varieties of berries, should absolutely be forbidden. Milk, on account of the fat and sugar it contains—besides its casein is difficult of digestion by many—should only be permitted in moderation. In short, generally speaking, the dietetic regimen best suited to these patients is a liberal allowance of albuminous matter, a diminution of the starches and fats, and a total abstinence from cane sugar.

Prohibit all forms of alcohol; it lessens molecular changes, and the elimination of excrementitious material favoring its accumulation in the blood. It also opposes the solution of uric acid in the blood, which favors its precipitation in the urine.

Pure water, which should be taken freely between meals, is the only diuretic permissible.

The treatment of secondary, or symptomatic stone, comes under the head of solvent remedies, which this paper will not permit of a consideration.

